# Kicking, Throwing, Grappling: How Combat Sports Shape Muscular Fitness and Motor Competence in Children

**DOI:** 10.3390/jfmk10010076

**Published:** 2025-02-25

**Authors:** Stevan Stamenković, Hrvoje Karničić, Jadranka Vlašić, Anja Topolovec, Damir Pekas

**Affiliations:** 1 Faculty of Sport and Physical Education, University of Nis, 18000 Nis, Serbia; stevajudo@yahoo.com; 2 Faculty of Kinesiology, University of Split, 21000 Split, Croatia; 3 Faculty of Kinesiology, University of Zagreb, 10000 Zagreb, Croatia; jadranka.vlasic@kif.unizg.hr (J.V.); anja.topolovec@kif.hr (A.T.); damir.pekas@kif.hr (D.P.)

**Keywords:** coordination, strength, motor skill, training, childhood

## Abstract

**Background/Objectives**: This study aimed to examine differences in motor competence and muscular fitness between children engaged in combat sports and their peers who do not participate in structured physical activity. **Methods**: The sample consisted of 120 healthy eight-year-old children, evenly divided into two groups: 60 children practicing combat sports (karate, judo, and wrestling) for at least one year and 60 children without structured sports involvement. Motor competence was assessed using the Test of Gross Motor Development-2 (TGMD-2), while muscular fitness was evaluated through standing broad jump, grip strength, 30 s sit-ups, bent arm hang, medicine ball throw, and push-ups. For differences between groups, the independent samples *t*-test was performed. **Results**: Results indicated that children practicing combat sports demonstrated significantly higher locomotor and manipulative skills (*p* < 0.01, ES = 0.76–1.25) and superior muscular fitness across all tests (*p* < 0.01, ES = 0.53–1.09) compared to their peers. **Conclusions**: These findings highlight the positive impact of combat sports on overall physical development, particularly in enhancing motor competence, muscle strength, and endurance. Given the critical role of motor competence and physical fitness at this age, integrating combat sports into daily routines can support long-term athletic development, encourage physical activity, and enhance overall health. Future research should explore the influence of specific combat sports on distinct physical attributes and consider additional factors such as total physical activity levels.

## 1. Introduction

Motor competence and physical fitness are essential components of healthy development in children, playing a crucial role in their ability to engage in physical activities, develop social skills, and maintain a good quality of life [1]. Participation in sports has been consistently associated with improved health-related quality of life, enhancing both physical and mental well-being through the development of motor skills, strength, endurance, and coordination [2]. Among various types of physical activity, combat sports stand out as an effective means to foster motor competence and physical fitness due to their emphasis on coordination, balance, and agility [3,4]. These sports not only support the physical aspects of development but also have psychosocial benefits, including increased self-discipline, resilience, and respect for others [5]. However, it is important to recognize that different combat sports may offer distinct benefits depending on their specific demands [5]. For example, judo emphasizes grip strength, dynamic balance, and throwing techniques, whereas boxing focuses more on upper-body coordination, reaction time, and cardiovascular endurance [6,7]. Wrestling, on the other hand, requires a combination of explosive strength, flexibility, and positional control [8]. Given these variations, future studies should consider examining the differential effects of various combat sports on children’s motor competence and physical fitness, rather than treating them as a homogeneous category.

An individual’s ability to efficiently and effectively perform various physical activities depends on both motor competence and physical fitness. Motor competence involves coordinating fine and gross motor skills, while physical fitness includes cardiovascular endurance, muscular strength, flexibility, and body composition, all of which contribute to overall health and well-being [9,10]. Sport and physical activity are essential for the development of motor competence and physical fitness in children. Early engagement in sports not only supports motor competence but also promotes physical fitness components such as strength, endurance, and flexibility, which are necessary for overall well-being [9,10]. Studies have shown that children engaged in multisport activities exhibit better motor coordination and higher levels of physical fitness than those who either focus on a single sport or do not participate in organized sports at all [11,12]. Similarly, children who explore a variety of sports tend to develop broader motor skills and are more physically fit than those who specialize early, highlighting the risks of overly focused training during developmental years [9]. However, while combat sports are often discussed in relation to coordination, balance, agility, self-discipline, and resilience, a more in-depth review of studies specifically linking combat sports to improvements in motor competence and muscular fitness is needed to clarify whether these sports provide unique benefits beyond the general advantages of physical activity [13].

Positive effects of participation in different sports was confirmed, underlining the importance of varied movement experiences for children’s motor development [10]. Accordingly, the balance between structured sports programs and unstructured play is crucial, as an excessive focus on skill-specific training may impede holistic motor development [14]. To maximize the physical and health benefits of sports, programs designed for children should prioritize diversity in activities and progressively integrate specific skills. Therefore, encouraging multisport participation during early childhood is crucial to optimize motor competence, physical fitness, and long-term health outcomes [12,15]. Furthermore, it is important to recognize how certain sports, such as combat sports, may encourage early specialization. Although they offer specific benefits in coordination and discipline, their intense focus on technique from a young age could limit the variety of movement experiences essential for holistic development.

While extensive research highlights the importance of motor competence and physical fitness for children’s development and health-related quality of life, significant gaps remain in understanding the nuanced effects of specific sports, particularly combat sports, on these aspects. Existing studies have established the benefits of multisport participation and structured physical activity in enhancing motor skills and physical fitness [2,4,12,14,15]. Nevertheless, there is limited evidence on how different forms of combat sports uniquely contribute to motor competence and muscular fitness compared to other types of physical activity.

Furthermore, while systematic reviews suggest the potential of martial arts to foster physical and psychosocial development [5], more targeted research is needed to determine the impacts of combat sports on specific outcomes in children. Addressing these gaps is crucial for designing evidence-based interventions and recommendations that leverage the full potential of combat sports for children’s comprehensive development. The aim of this study is therefore to compare the motor competence and muscular fitness of children engaged in combat sports with those not participating in structured physical activity. The hypothesis of this study is that children engaged in combat sports will demonstrate significantly better motor competence and muscular fitness compared to children who do not participate in structured physical activity.

## 2. Materials and Methods

### 2.1. Participants

To determine the sample size, the G*Power (Version 3.1.9.7, University of Dusseldorf, Germany) software was used with the assumed data (alpha = 0.05; beta = 0.80; coefficient of determination = 0.5) based on the results from [16]. The study sample consisted of 120 healthy children evenly divided into two groups based on their participation in physical activity. We used randomization, conducted with the tool available at www.randomizer.org, with participants assigned to groups by chance. In this way, we reduced bias and increased study validity. One group comprised 60 children engaged in combat sports training (22 girls), while the other included 60 children (20 girls) who did not participate in organized physical activities. All participants were 8 years old (mean age ± SD = 8.3 ± 0.5 years; height = 132.42 ± 5.32 cm; weight = 28.46 ± 5.38 kg). The combat sports group included children who regularly participated in structured training programs for karate, judo, and wrestling for at least one year, which was a key inclusion criterion. The non-organized activity group consisted of children who spent their free time in unstructured physical activities and had never been involved in formal sports training. Participation in the study was limited to healthy children who met the inclusion criteria. Exclusion criteria were if some children gave up during testing and children who did not have continuity in the training cycle (they gave up and continued with the same sport for a certain period of time). Parents were informed about the purpose and procedures of the research, and informed consent was obtained for their children’s involvement. Also, children were informed about the aim of this study and the testing protocol. The study was conducted in accordance with the Declaration of Helsinki and approved by the Ethics Committee of the Faculty of Sport and Physical Education, University of Nis (No. 04-428/2, approved 23 March 2024).

### 2.2. Testing Procedures

The testing was conducted at 9 am indoors in a school gymnasium with parquet flooring, under optimal conditions. Trained assistants from the Faculty of Sport and Physical Education, who had undergone prior training in standardized testing procedures, were engaged to administer the tests. The entire process was carried out according to a predetermined schedule and standardized protocols to ensure accurate and reliable results.

Prior to the testing, each participant completed a warm-up session lasting approximately ten minutes. The warm-up included light aerobic activities and mobility exercises to prepare the muscles and joints for physical exertion and to reduce the risk of injury. The warm-up protocol was the same on both days.

On the first day, testing focused on motor skills, which were assessed using the TGMD-2 test battery. After completing all motor skill tests, muscular fitness testing followed on the second day. This was conducted using age-appropriate and ability-specific standardized protocols and measures. All results were recorded individually for each participant, ensuring that every activity was performed under controlled conditions.

### 2.3. Testing

#### 2.3.1. Motor Competence

The Test of Gross Motor Development—Second Edition (TGMD-2) is one of the most widely utilized test batteries for assessing fundamental motor skills in children. It is a standardized tool designed to evaluate the motor skills of children aged 3 to 10 years. The TGMD-2 is applied to identify children who exhibit significant delays in motor skills compared to their peers, track individual progress in motor skill development, assess the effectiveness of motor programs, and serve as a research instrument for studies on motor skill development [17]. These authors also emphasize that the TGMD-2 test battery is highly reliable and valid.

According to Logan, Robinson, and Getchell [18], the TGMD-2 is specifically designed to measure skill development and is particularly suited for investigating the relationship between motor skills and participation in physical activities, as these skills form the foundation of many sports, including running, throwing, catching, and dribbling. Originally validated in 2000 [19], the TGMD-2 is based on normative data obtained from a study of 1208 children aged 3 to 10 years, conducted across 10 states in the United States. The test comprises 12 motor skills divided into two subtests. Each child performs each test twice, receiving a score of 1 for a correct execution and 0 if the skill is performed incorrectly. The estimated time to complete the full battery is approximately 10–20 min per child. The total raw score, calculated as the sum of all criteria for each subtest, ranges from 0 to 48 points. This score represents the most valuable outcome of the TGMD-2, as it reflects the key constructs assessed by the test and demonstrates high reliability. It serves as an accurate indicator of an individual’s current level of basic motor development. High scores indicate well-developed locomotor and manipulative skills.

The TGMD-2 test consists of two subtests designed to evaluate fundamental motor skills: locomotor skills and manipulative skills. These assessments follow a standardized procedure to ensure reliability and validity. Before testing, each task is explained and demonstrated to participants. Children are allowed one practice trial before formal testing begins, and if necessary, additional explanations and demonstrations are provided.

#### 2.3.2. Locomotor Skills

Running involves sprinting over a distance of 15.24 m, marked by two cones, with 3–4 m of space for deceleration beyond the second cone. The participant starts in a high stance, facing the second cone, and runs as quickly as possible after a signal. The task is performed twice, and the examiner, standing 3 m from the middle of the course, evaluates each attempt based on specific criteria.

Galloping is performed over a 7.62 m course, with two cones marking the path. Starting in a high stance, the participant gallops forward to the second cone and then gallops back. The task is repeated twice, and performance is scored based on adherence to the criteria.

Hopping assesses the participant’s ability to hop three times consecutively on one leg and then switch to the other leg. This is conducted on a 4.57 m non-slip surface. The participant begins in a straddle stance and hops on their dominant leg before repeating the task on the opposite leg. Each trial is scored individually.

Leaping involves crossing an obstacle, such as a beanbag placed between two parallel lines spaced 3.05 m apart, by running and leaping over it. Starting in a high stance behind one line, the participant leaps forward and lands cleanly. This is repeated twice, and the examiner scores each attempt from a 3 m distance.

Long jumping requires the participant to perform a standing broad jump from a marked line on a non-slip surface. Standing with feet together behind the line, the participant jumps forward with both feet as far as possible. Two trials are performed and scored based on execution.

Sliding evaluates lateral movement over a 7.6 m distance between two cones. The participant begins in a sideways stance behind the starting cone, performs a lateral gallop to the far cone, and slides back to the starting point. This is repeated twice, with performance scored based on adherence to the criteria [17].

#### 2.3.3. Manipulative Skills

Baseball strike involves striking a stationary lightweight ball (10.16 cm diameter) placed on a tee. The participant stands sideways to the tee in a straddle stance and strikes the ball forcefully with a plastic bat held in both hands. Two attempts are performed, and scoring is based on proper execution.

Dribbling requires the participant to dribble an age-appropriate ball (25.4 cm for children aged 3–5 years, standard basketball for ages 6–10) in place using one hand. Standing in a straddle stance, the participant dribbles the ball four consecutive times without moving their feet. Two trials are scored for proper technique.

Catching a ball assesses the participant’s ability to catch a lightweight ball thrown underhand in an arc by the examiner from a distance of 4.5 m. Only catches made between the waist and chest using both hands are scored. Each participant performs the task twice.

Kicking a ball evaluates the ability to kick a stationary ball placed on a beanbag toward a wall from a starting line 9.1 m away. The participant starts in a high stance, approaches the ball, and kicks it forcefully. Two trials are performed, with scoring based on successful execution.

Throwing a ball tests the participant’s ability to throw a small basketball forcefully toward a wall from a 6.1 m distance. Standing in a straddle stance, the participant uses their dominant hand to throw the ball. Each child performs the task twice, and scoring is based on execution.

Rolling a ball involves rolling a ball toward a wall, aiming to hit it between two cones set 1.2 m apart. The participant stands behind a line 7.6 m from the wall and uses their dominant hand to roll the ball. The task is performed twice, and performance is scored according to defined criteria.

For all tests, participants receive a score of 1 for each criterion met and 0 if the criterion is not met. Scores from both attempts are summed to calculate the final score for each skill [17].

#### 2.3.4. Muscular Fitness

The tests used for assessing muscular fitness in this study were used and validated for this age group [20,21]. The selected tests have been widely employed in numerous studies and have demonstrated strong methodological rigor, meeting fundamental psychometric standards. A study has demonstrated how these tests contribute to developing a Fitness Index model [22]. This model enables the early detection of health-related issues in large populations of children, providing an opportunity to plan appropriate interventions [22]. For this research, the following muscular fitness tests were utilized: standing broad jump, grip strength, 30-second sit-ups, bent arm hang, medicine ball throw, and push-ups.

#### 2.3.5. Standing Broad Jump

The standing broad jump test is performed indoors on a surface measuring at least 4 × 4 m. The testing equipment includes a mat marked with measurement increments and a measuring stick. The participant begins by standing with both feet behind a designated line, facing the mat. The task is to jump forward with both feet as far as possible. Each participant is given three attempts, with a 60 s rest between jumps. The longest valid jump of the three is recorded. A jump is considered invalid if the toes cross the starting line, the takeoff or landing is not done with both feet, a double hop or step is made during the jump, or the participant falls or sits upon landing. The final score is expressed in centimeters [23].

#### 2.3.6. Grip Strength

The grip strength test is conducted using a dynamometer suited and validated [24] for children (Takei Scientific Instruments Co., Tokyo, Japan). The participant stands upright, holding the dynamometer with their dominant hand, with their arm extended beside their body without bending the elbow. On the tester’s signal, the participant squeezes the dynamometer with maximum effort for a few seconds. Each participant completes two trials, and the best result is recorded. The grip strength score is measured in kilograms.

#### 2.3.7. 30-Second Sit-Ups

The 30 s sit-ups test is conducted using a mat on the floor. The participant lies on their back with their knees bent at a 90-degree angle and feet hip-width apart, while an assistant secures their feet to the ground. Arms are crossed over the chest with hands touching opposite shoulders. At the tester’s signal, the participant raises their torso to touch their elbows to their thighs and then returns to the starting position. This sequence is repeated as quickly as possible within 30 s. Improperly executed sit-ups, such as lifting feet off the ground or failing to meet the correct elbow–thigh position, are not counted. The score is the total number of correctly performed sit-ups in 30 s [25].

#### 2.3.8. Bent Arm Hang

The bent arm hang test is performed on a bar set at an appropriate height. The participant uses an underhand grip (palms facing them) to hold the bar, with hands shoulder-width apart. With assistance, the participant is lifted into the starting position, with the chin above the bar and the body extended and stable. Time begins when the participant holds this position independently and ends when the chin drops below the bar or contacts it. The total duration of the hang is measured in seconds [26].

#### 2.3.9. Medicine Ball Throw

The medicine ball throw involves pushing a 1 kg medicine ball as far as possible using both hands. The participant begins in a standing position with feet parallel and shoulder-width apart, holding the ball against their chest. From this position, they push the ball forward forcefully without stepping or lifting their feet. Each participant performs two throws, and the longer distance is recorded. The final score is measured in meters [27].

#### 2.3.10. Push-Ups

The push-ups test measures the number of correctly executed push-ups completed until exhaustion. The participant begins in the standard push-up position with hands placed slightly wider than shoulder-width apart, fingers pointing forward, back straight, and legs fully extended. Each push-up consists of lowering the body until the upper arms reach a 90-degree angle at the elbows and then fully extending the arms. The test ends after the second improper push-up, which can include failing to achieve a 90-degree angle, arching the back, or pausing for an extended time. The total number of valid push-ups completed is recorded [28].

### 2.4. Statistical Analysis

The statistical analysis was conducted using SPSS software, version 24.0. Descriptive statistics were calculated for all variables. The Kolmogorov–Smirnov test was used to assess the normality of data distribution. To examine differences in muscular fitness and motor competence between children engaged in combat sports and those not participating in organized physical activity, an independent samples *t*-test was performed. Means and standard deviations for independent variables were used to calculate effect sizes (ES; Hedge’s g) for each variable in both groups. Effect size values, accompanied by 95% confidence intervals, were interpreted using the following scale: <0.2—trivial; 0.2–0.6—small; >0.6–1.2—moderate; >1.2–2.0—large; >2.0–4.0—very large; >4.0—extremely large [29].

## 3. Results

**Figure 1:**
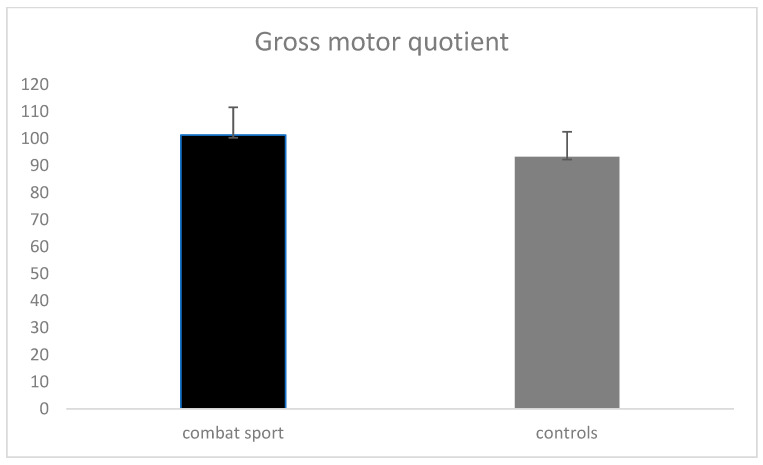
Gross motor quotient in combat group and controls.

The results in Table 1 indicate significant differences in motor competence measures between children engaged in combat sports and their peers. Children in combat sports scored significantly higher in locomotor skills (mean = 11.54 ± 2.03) compared to controls (mean = 9.09 ± 1.77), with a mean difference of 2.54 and a *p*-value of 0.01. The effect size (ES) was large at 1.25, suggesting a substantial practical significance in favor of combat sports participants. They also outperformed their peers in manipulative skills, with a mean score of 10.89 ± 1.80 versus 9.54 ± 1.62. The mean difference was 1.35, with a *p*-value of 0.01, reflecting statistical significance and a moderate effect size (ES = 0.76). Additionally, the gross motor quotient (Figure 1) was higher among combat sports participants (mean = 101.28 ± 10.28) than controls (mean = 93.23 ± 9.27). The mean difference was 8.03 (95% CI: 4.49, 11.57), with a *p*-value of 0.01, indicating statistical significance and a moderate to large effect size (ES = 0.82).

Figure 1 shows a comparison of the gross motor quotient between individuals involved in combat sports and a control group. The graph suggests that individuals involved in combat sports may have a higher gross motor quotient compared to the control group. These data visually indicate a potential positive impact of combat sports on gross motor skills.

The results in Table 2 reveal significant differences in muscular fitness between children engaged in combat sports and their peers, with combat sports participants consistently outperforming the control group. For the standing broad jump, children in combat sports achieved a greater distance (mean = 145.14 ± 15.27 cm) compared to controls (mean = 136.21 ± 16.34 cm), with a mean difference of 8.89 cm (95% CI: 3.17, 14.61) and a *p*-value of 0.01 (moderate effect size ES = 0.56). In grip strength, combat sports participants showed significantly higher (*p* = 0.01) performance (mean = 25.86 ± 4.51 kg) compared to controls (mean = 23.11 ± 5.84 kg), with a mean difference of 2.76 kg and a moderate effect size (ES = 0.53). The 30 s sit-up test also favored combat sports participants, with a mean difference of 4.66 (95% CI: 3.13, 6.19) and a *p*-value of 0.01 (large effect size ES = 1.09). For the bent arm hang, combat sports participants held for a significantly longer (*p* = 0.01) duration (mean = 25.45 ± 10.75 s) than controls (mean = 15.74 ± 13.12 s), with a mean difference of 9.71 s and a large effect size (ES = 0.80). Similarly, combat sports participants performed better (*p* = 0.01) in the medicine ball throw (mean = 3.84 ± 0.55 m) compared to controls (mean = 3.42 ± 0.43 m), with a large effect size (ES = 0.85). Finally, in push-ups, combat sports participants completed more repetitions (mean = 16.25 ± 5.30) compared to controls (mean = 12.49 ± 4.46), with a mean difference of 3.76 repetitions (95% CI: 1.99, 5.53) and a *p*-value of 0.01.

## 4. Discussion

The aim of this research was to examine the differences in motor and muscular fitness between children engaged in combat sports and their peers who are not involved in such activities. The findings of the study demonstrated significant differences favoring children participating in combat sports across all measured variables. Specifically, these children exhibited superior locomotor and manipulative skills, as well as higher gross motor proficiency compared to their peers. Furthermore, they outperformed the control group in all measures of muscular fitness, including standing broad jump, grip strength, sit-ups, bent arm hang, medicine ball throw, and push-ups. The results of this study support the hypothesis, as children engaged in combat sports demonstrated significantly better motor competence and muscular fitness compared to those who did not participate in structured physical activities.

The findings of this study, which highlight superior motor competence in children engaged in combat sports compared to their peers, align with previous research emphasizing the relationship between sports participation and motor skill development. Utesch et al. [1] showed a strong correlation between motor competence and physical fitness from early childhood to early adulthood, which supports our observation that combat sports improve motor proficiency. Rodrigues et al. [4] also noted that martial arts and combat sports play a key role in developing coordination and motor skills, which are essential for overall motor competence. Moreover, Sadowski [3] identified coordination motor abilities as dominant in combat sports, likely explaining the observed advantage in locomotor and manipulative skills among our participants. The results are consistent with studies such as Stamenković et al. [5], which documented significant benefits of martial arts in improving motor skills among children. In comparison to broader sports activities, Popović et al. [11] reported that multisport participants showed improved gross motor coordination, but children in sport-specific activities, such as combat sports, may receive tailored training that directly targets and refines these skills, potentially leading to the differences observed in our study.

The observed superiority in muscular fitness among children engaged in combat sports is supported by previous findings emphasizing the physical benefits of sports participation. Fransen et al. [9] provides valuable comparative insights into how participation in structured sports influence physical fitness, supporting our findings on the benefits of structured combat sports training on standing broad jump, handgrip strength, and sit ups. Although the authors focused on sport sampling versus specialization, their findings support the idea that structured sports training positively influences physical fitness, which aligns with our results showing superior performance in combat sports participants. This is likely due to the intensity and functional demands of combat sports, which include dynamic and static strength training components. Stanković et al. [12] also highlighted improved motor coordination in children participating in multisport activities; however, sports involving diverse and intensive movements, such as combat sports, might yield even greater muscular fitness improvements due to their high-frequency engagement of upper and lower body muscles [2]. The findings of this study are further corroborated by Opstoel et al. [10], who reported higher physical fitness levels in children participating in sports emphasizing strength and power. The explosive and repetitive movements inherent in combat sports, combined with high levels of engagement in skill-specific tasks [14], may explain the significant advantages observed in muscular fitness among our participants. These activities likely encourage neuromuscular adaptations and foster higher levels of physical preparedness compared to less intensive recreational activities.

Combat sports and martial arts have long been recognized for their potential to enhance motor competence and muscular fitness in children. Woodward [30] highlights the broad health benefits of martial arts, including improved physical conditioning and motor skills. Specifically, coordination motor abilities, essential in combat sports like judo, are noted to improve with age and practice intensity, as shown by Sterkowicz, Lech, and Jaworski [31]. These findings align with Sadowski [3], who identified coordination as a dominant motor ability developed through combat sports due to their dynamic and multifaceted movements. Such activities demand balance, agility, strength, and precision, all contributing to better locomotor and manipulative skills, as observed in our study. Additionally, Sterkowicz-Przybycień, Kłys, and Almansba [32] emphasized the educational and behavioral benefits of martial arts in preschool-aged children, which could extend to the development of physical capabilities as these children age. Rodrigues, Marttinen, and Banville [4] further consolidate these findings, noting in their 10-year review that martial arts and combat sports effectively promote muscular fitness and skill development in youth. Our results support these assertions, as children practicing combat sports demonstrated superior motor competence and muscular fitness compared to their peers, reinforcing the critical role of such activities in fostering holistic physical development. It is important to consider that while combat sports may offer significant benefits for motor competence and muscular fitness, they also carry an increased risk of injury, which could potentially outweigh some of the physical benefits, especially in high-contact disciplines [13]. The study’s results are useful for promoting the inclusion of combat sports in physical education and youth sports programs to enhance motor competence and muscular fitness in children. Additionally, they can guide educators, coaches, and policymakers in designing targeted training programs that support long-term physical development and encourage healthy, active lifestyles from an early age.

While most differences between groups were statistically significant, the effect sizes were moderate or small, suggesting that the practical impact may be limited. One possible explanation for these smaller effect sizes is the relatively high standard deviation within each group, indicating variability in individual performance. Additionally, while combat sports training likely enhances muscular fitness, the absolute difference in grip strength may not translate into meaningful advantages in everyday activities for children. However, even moderate improvements in strength and power can be beneficial in the long-term development of athletic performance and health promotion, particularly as children progress in their training.

Some limitations should be mentioned in the current study. One limitation of this study is that we did not measure the participants’ overall leisure time physical activity outside of their engagement in combat sports. This could have influenced the observed differences in motor and muscular fitness, as additional physical activities might have contributed to the participants’ physical development. One of the limitations of this study is the lack of consideration for baseline differences, such as socioeconomic status, access to resources, and initial fitness levels, all of which could potentially influence the results. These factors were not controlled for or accounted for in the analysis, and ideally, they should have been considered to ensure that any observed differences in outcomes are not influenced by these underlying variables. Furthermore, the relatively small sample size limited our ability to analyze differences between specific combat sports separately. This may have masked potential sport-specific variations in motor competence and muscular fitness, as different combat sports could have unique effects on these outcomes based on their training focus and demands. Future research with larger and more diverse samples, including measures of total physical activity, could provide a more comprehensive understanding of the impact of combat sports on children’s physical development.

## 5. Conclusions

This study demonstrated significant differences in motor competence and muscular fitness between children engaged in combat sports and their peers who do not participate in organized physical activity. Children involved in combat sports exhibited superior locomotor and manipulative skills, as well as greater muscular fitness. These findings emphasize the role of combat sports in enhancing both fundamental motor skills and muscular fitness, suggesting that such activities provide a comprehensive foundation for physical development during childhood.

The results of this study highlight the importance of structured combat sports training as a valuable approach to improving physical fitness and motor competence in children. These findings can inform educators, coaches, and policymakers about the benefits of including combat sports in school and extracurricular programs. Practical applications for teachers can implement structured, age-appropriate combat sports programs focusing on motor skills, strength, and progression, while fostering a safe and inclusive environment with regular physical education curricula. Additionally, practitioners working with youth can use this information to design tailored training regimens that foster motor development and enhance muscular fitness. Parents can support their children’s participation by encouraging engagement, collaborating with coaches, and promoting a balanced, active lifestyle through multisport activities. Future initiatives should aim to promote the accessibility of combat sports while also encouraging their incorporation into broader physical activity strategies to support healthy growth and development in children.

## Figures and Tables

**Table 1:** Difference in motor competence between children engaged in combat sports and their peers.

	Combat Sports	Controls	Mean Diff (CI)	*p*	ES (CI)
Locomotor skills	11.54 ± 2.03	9.09 ± 1.77	2.54 (1.74, 3.16)	0.01	1.25 (0.86, 1.64)
Manipulative skills	10.89 ± 1.80	9.54 ± 1.62	1.35 (0.71, 1.99)	0.01	0.76 (0.39, 1.13)
Gross motor quotient	101.28 ± 10.28	93.23 ± 9.27	8.03 (4.49, 11.57)	0.01	0.82 (0.44, 1.19)

Values are mean ± SD; significant difference at *p* < 0.05.

**Table 2:** Difference in muscular fitness between children engaged in combat sports and their peers.

	Combat Sports	Controls	Mean Diff (CI)	*p*	ES (CI)
Standing broad jump (cm)	145.14 ± 15.27	136.21 ± 16.34	8.89 (3.17, 14.61)	0.01	0.56 (0.19, 0.92)
Grip strength (kg)	25.86 ± 4.51	23.11 ± 5.84	2.76 (0.87, 4.65)	0.01	0.53 (0.16, 0.89)
30 s sit-ups (n)	23.50 ± 3.82	18.84 ± 4.63	4.66 (3.13, 6.19)	0.01	1.09 (0.71, 1.47)
Bent arm hang (s)	25.45 ± 10.75	15.74 ± 13.12	9.71 (5.37, 14.05)	0.01	0.80 (0.43, 1.18)
Medicine ball throw (cm)	3.84 ± 0.55	3.42 ± 0.43	0.42 (0.24, 0.60)	0.01	0.85 (0.47, 1.22)
Push-ups (n)	16.25 ± 5.30	12.49 ± 4.46	3.76 (1.99, 5.53)	0.01	0.76 (0.39, 1.13)

Values are mean ± SD; significant difference at *p* < 0.05.

## Data Availability

Data are available upon reasonable request.
